# An mRNA-LNP vaccine expressing TP0435 provides protective immunity in rabbits against *Treponema pallidum* challenge

**DOI:** 10.1080/22221751.2026.2685927

**Published:** 2026-06-21

**Authors:** Zhiyu Lu, Yizhou Lu, Di Liu, Fangzhi Du, Qingyun Wu, Guoyang Liao, Yanan Wu, Rui-Li Zhang, Jian Zhou, Qian-Qiu Wang

**Affiliations:** a Hospital for Skin Diseases, Institute of Dermatology, Chinese Academy of Medical Sciences & Peking Union Medical College, Nanjing, People’s Republic of China; b Institute of Medical Biology, Chinese Academy of Medical Sciences and Peking Union Medical College, Kunming, People’s Republic of China; c Department of Dermatology, The Second Affiliated Hospital of Nanjing Medical University, Nanjing, People’s Republic of China

**Keywords:** Syphilis, *Treponema pallidum*, TP0435, mRNA-LNP vaccine, immunoprotection

## Abstract

Syphilis, a sexually transmitted disease caused by *Treponema pallidum* (*T. pallidum*), is a major public health concern globally. Although syphilis can be easily diagnosed and treated with an inexpensive antibiotic, it continues to be a significant global health problem. Vaccination is the most cost-effective public health intervention to prevent and control infections. Therefore, a safe and effective syphilis vaccine is urgently needed. *T. pallidum* surface exposed antigens are regarded as the most promising vaccine candidates. In recent years, mRNA-LNP (lipid nanoparticles) vaccines also showed promising protective results for bacterial diseases. TP0435, encoding the highly immunogenic *T. pallidum* 17-kDa lipoprotein, is a periplasmic antigen that has also been shown on the pathogen surface. In this study, we successfully constructed the TP0435 mRNA-LNP vaccine, which was stable, effectively expressed, and exhibited no acute toxicity. In BALB/c mice, a 5 µg dose elicited strong antigen-specific humoral responses, Th1-biased cellular immunity, and enhanced CD4^+^ central memory T-cell formation. In New Zealand White (NZW) rabbits, both TP0435 mRNA-LNP and protein vaccines delayed lesion formation and reduced *T. pallidum* burden; moreover, the mRNA-LNP vaccine provided superior protection, completely preventing ulcer formation and showing lower *T. pallidum* loads at lesion sites than the protein vaccine, while effectively limiting pathogen dissemination. Histopathological analysis revealed that, at nodular lesions, the mRNA vaccine group exhibited reduced neutrophil infiltration and increased proportions of macrophages and lymphocytes compared with ulcerative lesions in the LNP group. Therefore, we successfully constructed an mRNA-LNP vaccine targeting TP0435, which might be a promising syphilis vaccine candidate.

## Introduction

Syphilis is a chronic systemic sexually transmitted disease (STD) caused by *Treponema pallidum* (*T. pallidum*), which causes damage to multiple organs and tissues and increases the risk of infection with other STDs. The first cases of this disease were documented in Europe in the late fifteenth century [1]. A pivotal breakthrough came in 1905, when Schaudinn and Hoffmann identified the bacterial pathogen responsible for this venereal infection. Decades later, in 1943, another milestone was achieved when the first cases of syphilis were successfully treated with penicillin [2]. According to data from the World Health Organization (WHO), an estimated 19.9 million people were living with syphilis globally in 2016, with approximately 6 million new cases reported annually and more than 107,000 attributable deaths [3]. A high prevalence of syphilis is observed in low-income and middle-income countries, and it has been rising in high-income countries over the past few decades. Notably, the upward trend is most prominent among men who have sex with men (MSM) [4].

Although syphilis is curable with benzathine penicillin, challenges in syphilis treatment persist due to penicillin allergies and the emergence of macrolide-resistant strains [5]. Vaccination is the most cost-effective public health intervention to prevent and control infections and may even completely eliminate diseases. Therefore, research on syphilis vaccines is of great necessity. An effective syphilis vaccine should meet the following requirements. First of all, due to the variability among *T. pallidum* strains, vaccine targets should be selected from the conserved regions of spirochete surface antigens to induce the body to acquire cross-strain immune protection. Secondly, the vaccine should effectively induce the body to generate T helper 1 (Th1)-type immune responses and opsonizing antibodies, promote the phagocytosis of macrophages, and accelerate the clearance of *T. pallidum*. Thirdly, the syphilis vaccine should induce the body to produce complete protective immunity and prevent the dissemination of *T. pallidum* in the body and the progression of the disease [6].

To date, existing traditional *T. pallidum* vaccines mainly included inactivated vaccines, attenuated vaccines, subunit vaccines and DNA vaccines [7, 8]. Inactivated vaccines exhibit high safety and provide comprehensive protection, but they require multiple injections. Attenuated vaccines demonstrated strong immunogenicity and induced robust humoral and cellular immunity, but they carried the risk of reverting to high virulence after administration. Recombinant subunit vaccines were made from specific surface structural components (antigens) of *T. pallidum*, which contained no nucleic acid. Subunit vaccines could prevent the body from generating antibodies irrelevant to the pathogen, thereby reducing vaccine-associated adverse reactions. However, subunit vaccines had low immunogenicity and required combination with immunological adjuvants to achieve favourable immune responses. Existing studies have found that although DNA vaccine immunization could induce the production of high levels of antibodies, the cellular immune response was relatively weak, resulting in a suboptimal immune effect [9]. Recently, an mRNA-LNP (lipid nanoparticles) targeting TP0136-derived T-cell epitope was first reported and elicited potent cellular immunity, controlled treponemes, and prevented ulcers, indicating the great potential of mRNA-LNP vaccines in syphilis [10].

mRNA vaccines represent the third generation of vaccines, emerging in the 1990s and rapidly developing in a record-breaking time after COVID-19 pandemic [11]. The foreign mRNAs encoding antigens are introduced into the cytoplasm to synthesize antigens and further induce the immune response. This is achieved by using a delivery system such as LNP to transport the synthetic mRNA into cells [11]. mRNA vaccines have several important advantages compared to traditional vaccines. First of all, the safety of mRNA vaccines is high. The explanation is that mRNA does not integrate with the host DNA and is non-infectious. Secondly, the efficacy of mRNA vaccines is high, as modifications in the mRNA structure can make the vaccine more stable and effective, with reduced immunogenicity. In addition, mRNA vaccines have a high production capacity. mRNA vaccines are produced in a cell-free environment, hence allowing rapid, scalable, and cost-effective production [11].

So far, mRNA vaccines continue to demonstrate success against viral diseases and tumours, The development of mRNA vaccines against bacterial diseases has been more challenging [12]. Only a limited number of publications have shown protective efficiency of mRNA vaccines against bacteria. To date, the targeted bacterial pathogen of mRNA vaccines that underwent a preclinical evaluation mainly included *Chlamydia trachomatis, Y. pestis*, *L. monocytogenes*, *S. pyogenes* and *S. agalactiae*, *P. aeruginosa* and *S. typhimurium*. mRNA vaccines targeting *B. burgdorferi* and *M. tuberculosis* were under clinical evaluation [13]. Unlike viruses, bacteria can synthesize proteins on their own. However, protein synthesis in human cells undergoes post-translational modifications, which leads to structural differences between the proteins expressed by mRNA in the human body and those produced by bacteria, affecting their stability and immunogenicity. Additionally, bacteria have much larger genomes and far more complex structures compared with viruses. Structural elements of bacteria can generate thousands of antigens, making it extremely difficult to identify effective antigens suitable for vaccine development. Moreover, common bacterial diseases are usually not severe infectious diseases, do not usually spread on a large scale in a short time, and can be treated with highly effective and low-cost antibiotics.

TP0435, also designated as Tpp17, is identified as a major antigen of *T. pallidum* and exhibits substantial utility in the serological diagnosis of syphilis. TP0435 was also considered to be a periplasmic lipoprotein that was shown on the pathogen surface with adhesin function [14]. Such evidence indicated that TP0435 might also be a possible vaccine candidate. Parveen et al. [15] reported that rabbits immunized with the TP0435-expressing *Borrelia burgdorferi* B31HP strain effectively stimulated both humoral and cellular immunity. However, the lesion progression and *T. pallidum* burdens in lesion sites were not reduced in rabbits immunized with the TP0435-expressing *Borrelia burgdorferi* B31HP strain compared with the controls**.** To better examine the protective effect of TP0435, we constructed a novel mRNA vaccine and compared the immunogenicity and protection with recombinant TP0435 protein against *T. pallidum* Nichols strain.

## Methods

### Ethics statement

This study was carried out strictly according to the Animal Ethics Procedures and Guidelines of the People’ s Republic of China. The experimental protocols were approved by the Ethics Committee of the Institute of Dermatology, Chinese Academy of Medical Sciences (ethical approval number 2025-DW-058).

### Design of mRNA constructs

The full length of TP0435 sequence was obtained from GenBank. Plasmid corresponding to the sequence was amplified using E. coli Stabl3 (ThermoFisher Scientific, Waltham, MA, USA). Following plasmid extraction, linearized template was generated via digestion with the BspQI restriction enzyme and purified using DNA magnetic beads (Vazyme, Nanjing, China). Subsequently, in vitro transcription (IVT) was performed to synthesize messenger RNA (mRNA), using T7 RNA polymerase (Vazyme, Nanjing, China), CleanCap (Syngenbio, Nanjing, China), and deoxyribonucleoside triphosphates (dNTPs) – where uridine triphosphate (UTP) was substituted with m¹ψ−5′ triphosphate (Syngenbio, Nanjing, China). After completion of the IVT reaction, the synthesized mRNA was purified using RNA magnetic beads (Vazyme, Nanjing, China). An ultraviolet spectrophotometer quantified mRNA concentration and formaldehyde-denaturing agarose gel electrophoresis assessed mRNA integrity and purity.

### LNP preparation and characterization

The prepared mRNA solutions were encapsulated in LNP. mRNA solutions were formulated at a concentration of 200 μg/mL in 25 mM sodium acetate buffer (pH 5.5). The LNP formulation comprised four components: cationic lipid, phosphatidylcholine, cholesterol, and polyethylene glycol (PEG)-lipid, at a molar ratio of 50:10:38.5:1.5, which were dissolved in anhydrous ethanol. mRNA and LNP components were mixed at a 3:1 flow ratio using a microfluidic device to form an mRNA-LNP mixture. Subsequently, ethanol was removed and replaced with 25 mM Tris-HCl buffer (pH 7.5) using a 100 kDa ultrafiltration centrifugal filter, and the solution was concentrated to produce the mRNA-LNP vaccine. Encapsulation efficiency was determined using the Quant-iT™ RiboGreen RNA Kit (ThermoFisher Scientific, Waltham, MA, USA), while particle size and polydispersity index were measured with a Nanoparticle Size and Zeta Potential Analyzer (Malvern Panalytical, Malvern, UK).

### Cell culture, transfection, and expression of TP0435 mRNA by western blot

HEK293 T cells were cultured in Dulbecco’s modiﬁed Eagle’s medium (DMEM; Gib co) with 100 U/mL penicillin-streptomycin (Thermo Fisher, Waltham, MA, USA) and 10% fetal bovine serum (Thermo Fisher, Waltham, MA, USA). Cells were cultured at 37 ℃ in a humidified 5% CO_2_ atmosphere. TP0435 antigen expression was detected by western blot analysis. Following the manufacturer’s protocol, HEK293 T cells were transfected with 2 µg of mRNA using Lipofectamine 3000 (Vazyme, Nanjing, China). Then, the cells were cultured for 48 h at 37℃ in a humidified 5% CO_2_ atmosphere. Cells were then collected, washed with PBS (pH 7.4), and lysed by radioimmunoprecipitation assay (RIPA) lysis buffer (Beyotime, China) supplemented with 1 mM phenylmethylsulfonyl fluoride (PMSF) (P0100, Solarbio, China) in loading buffer for Western blot analysis. The total protein was separated on 4−20% Bis-Tris gradient precast gels (GenScript, USA) at 120 V for 80 min and electro-transferred to polyvinylidene diﬂuoride (PVDF) membranes at 400 mA for 20 min. Membranes were then treated with blocking buffer (Bio-Rad, USA) for 10 min and incubated with primary antibodies at 4℃ overnight, followed by incubation with antirabbit IgG and HRP-linked antibody (Cell Signaling, USA) at 37℃ for 1 h. These membranes were treated with chemiluminescence chemicals (Affinity, USA), and the images were acquired on the ChemiDocTM MP Imaging System (BIO-RAD).

#### Preparation of the TP0435 recombinant protein

Plasmids were transformed into E. coli BL21(DE3) competent cells, plated, and incubated overnight at 37℃. For expression screening, single colonies were inoculated into 5 mL LB medium and grown at 37℃ followed by induction with 0.5 mM IPTG for 4 h. Cells were harvested by centrifugation, and expression was analysed by SDS-PAGE and Western blot. For expression optimization, four cultures were induced with IPTG at final concentrations of 0.2 mM or 1 mM and incubated at 37℃ or 15℃ with shaking (220 rpm) for 4 or 16 h, respectively; soluble and insoluble fractions were prepared from harvested cells and analysed by SDS-PAGE. For large-scale expression, the optimal clone was cultured in 1 L LB induced with IPTG at 37°C for 4 h, harvested by centrifugation, and resuspended for ultrasonic cell disruption. The lysate was centrifuged, and the supernatant was applied to Ni-NTA affinity chromatography (equilibrated with PBS-NaCl, pH 7.4; washed with PBS-NaCl containing 50 mM imidazole; eluted with PBS-NaCl containing 500 mM imidazole). Purified protein fractions were analysed by SDS-PAGE, pooled, dialyzed against PBS (pH 7.4), and sterilized by filtration.

### Mouse immunization

Female BALB/c mice (6–8 weeks old, specific-pathogen-free) were used in this study, supplied by GemPharmatech Co., Ltd. (Nanjing, China). BALB/c mice are commonly used for immunogenicity evaluation due to their well-characterized immune background and reproducible Th1/Th2 responses. Female mice are frequently selected in immunogenicity studies because they generally exhibit more stable immune responses and reduced aggression-related variability when group-housed [10, 16, 17]. Four groups were established by random allocation, with 10 mice per group. Mice were intramuscularly injected with TP0435 mRNA-LNP vaccine at different doses (1, 5, 10 μg) into the hind limb. For the LNP group, mice received an equivalent volume of LNPs alone. Primary immunization was administered on day 0, with a booster dose given on day 14. On day 28, five mice from each group were sacrificed, and blood was collected.(additional samples were collected on day 14), and major organs were harvested. The remaining mice were sacrificed on day 56. Blood samples and spleens were also collected on day 56. The mouse immunization experiments were designed primarily to evaluate the immunogenicity of the TP0435 mRNA-LNP vaccine, as there have been no prior studies of full-length *T. pallidum* protein-based mRNA-LNP vaccines. At this stage, our focus was to establish the feasibility and immunogenic profile of the mRNA-LNP platform. We performed subsequent immunization experiments in New Zealand rabbits, where both TP0435 mRNA-LNP and protein vaccines were evaluated for immunogenicity and protective efficacy.

### Rabbit immunization

Male New Zealand White (NZW) rabbits (8–13 weeks old, weighing 2.5–3.0 kg) were used in this study. The rabbit challenge model represents the gold-standard model for *T. pallidum* infection, as rabbits are the only small animal species that reliably develop characteristic orchitis and cutaneous lesions following intradermal inoculation. Male New Zealand White rabbits are traditionally used because they develop consistent and quantifiable orchitis after intratesticular challenge, which provides a well-established and reproducible readout for protection efficacy [18–20]. The rabbits were randomly assigned to three groups with 8 animals per group. Animals in the TP0435 mRNA-LNP group received an intramuscular injection of 5 μg of the corresponding mRNA-LNP vaccine into the hind limb . Animals in the TP0435 protein vaccine group received an intramuscular injection of a 1:1 mixture of the 100 μg dose of TP0435 full-length recombinant protein and TiterMax Gold Adjuvant. The LNP group was administered an equivalent volume of LNPs via the same route. Primary immunization was performed on day 0, with a booster immunization given on day 14. Blood samples were collected from the ear veins on days 14 and 28. On day 28, five rabbits from each group were randomly euthanized, and their spleens were collected for splenocyte culture. The remaining rabbits were retained for subsequent *T. pallidum* challenge experiments.

### Syphilis serological analysis

Sera were collected from NZW rabbits at 2 weeks after primary and booster immunization, and the Treponema pallidum Particle Agglutination assay (TPPA), Gold Immunochromatographic Assay (GICA), and Rapid Plasma Reagin test (RPR) were performed according to the manufacturers’ instructions.

### *T. pallidum* challenge procedure

Two weeks following the booster immunization, the immunized rabbits were sedated. Their dorsal fur was shaved, and the exposed skin was disinfected with 75% ethanol. The *T. pallidum* challenge model used in this study was also established based on previously reported classical experimental models [18–20]. Each rabbit was intradermally challenged at 8 distinct sites: at each site, 0.1 mL of a suspension containing 1 × 10⁶ freshly isolated *T. pallidum* Nichols strain (prepared in 0.9% saline) was administered. *T. pallidum* Nichols strain was propagated in healthy male NZW rabbits via intratesticular inoculation and harvested as previously described by Lukehart et al. [21]. The Nichols strain employed in this study was an original liquid nitrogen-preserved stock (P0), which was revived and propagated through intratesticular passage in New Zealand white rabbits for 2–3 generations (P2–P3) prior to use. All passages were harvested at peak infection (10-14 days post-inoculation) to ensure high bacterial burden and stable virulence. Immediately after harvest, treponemes were examined under dark-field microscopy to assess characteristic spiral morphology and active motility, and organisms were enumerated to standardize the inoculum. Only preparations demonstrating robust motility were used for challenge. This passage and harvesting protocol follow the classical and widely accepted procedures for experimental syphilis models and is intended to maintain consistent virulence and ensure reproducibility of the challenge experiment. The development of lesions at the challenge sites was recorded and photographed daily. Additionally, the diameters of each lesion were measured daily. On day 21, all rabbits were euthanized, and the skin lesions and organs were harvested for further experiments.

### Safety of mRNA-LNP vaccine

Rectal temperature and body weight of mice were measured for 7 days after primary immunization. Blood samples were collected from each group at 4 weeks following the first immunization. Serum was then separated after centrifugation. Serum biochemical indicators were automatically measured using an automatic biochemistry analyzer (Chemray 800, China). Two weeks post the final immunization, all mice were euthanized; the organs were harvested, fixed, embedded in paraffin, and sectioned for histopathological analysis. Following conventional hematoxylin and eosin (H&E) staining, the tissue sections were examined under a light microscope.

### Analysis of speciﬁc antibody levels

The speciﬁc antibody levels were determined by indirect ELISA. The 96-well plates were coated with 1 μg/mL purified recombinant TP0435 protein. Excess antigen was washed off with PBST. Then 200 μL of blocking buffer (1 g BSA dissolved in 50 mL PBS) was added and incubated at 37°C for 2 h. Then the wells were washed three times with PBST. Mouse and rabbit serum were two-fold serially diluted starting from a dilution of 1:100. After 1 h of incubation at 37℃, the wells were washed three times with PBST again. Horseradish peroxidase (HRP)-conjugated goat anti-mouse IgG (1:10,000) or HRP-conjugated goat anti-rabbit IgG (1:10,000) was subsequently added to the wells and incubated at 37℃ for 1 h. The plates were washed three times with wash buffer, and 100 μL of TMB (3,3′,5,5′-tetramethylbenzidine) peroxidase substrate was added per well and incubated at 37℃ for 15 min. Then 100 μL stop solution was added to stop the colour reaction. The absorbance at 450 nm (A450 value) was detected with a microplate reader (Infinite 200 Pro, Switzerland). Two-fold serial dilutions of serum were made and the endpoint titer was considered to be the last serum dilution with readings higher than the 2.1-fold of the negative controls.

### Preparation of splenocyte suspension

Two weeks post the final immunization, mice were euthanized, and their spleens were harvested for the preparation of splenic cell suspensions. Two weeks after the final immunization, five rabbits in each group were randomly euthanized, and their spleens were harvested. Each spleen was homogenized through a 200-mesh cell strainer to obtain a splenic cell suspension. The suspension was centrifuged at 2000 rpm for 10 min. Following centrifugation, red blood cell (RBC) lysate was added to lyse RBCs, and the mixture was incubated for 5 min. Phosphate-buffered saline (PBS) was then added to terminate the lysis reaction. After another centrifugation step (2000rpm, 5 min), PBS was added to wash the splenic cell suspension. A third centrifugation (2000rpm, 5 min) was performed, and the resulting splenocytes were resuspended in RPMI-1640 medium supplemented with 10% fetal bovine serum (FBS), penicillin (100 U/mL), and streptomycin (100 μg/mL) for subsequent detection assays.

### Measurement of Th1, Th2, and Th17-associated cytokines in splenocyte culture supernatants from mice and rabbits

Mouse and rabbit splenic lymphocytes collected as described above were seeded in 6-well culture plates at a density of 1 × 10⁷ cells per mL. Each well was treated with 10 μg/mL recombinant protein, with PBS serving as the blank control; the plates were then incubated at 37℃ for 24 h. After incubation, cell culture supernatants were harvested to determine the secretion levels of interferon-γ (IFN-γ), tumour necrosis factor-α (TNF-α), interleukin-2 (IL-2), interleukin-12 (IL-12), interleukin-4 (IL-4), interleukin-10 (IL-10), and interleukin-17A (IL-17A), from mouse and rabbit splenocytes. Assays were performed according to the manufacturer’s instructions for the respective ELISA kits (Elabscience, China; Ruisaiqi Biotechnology, China). The absorbance at 450 nm was measured using a microplate reader (Infinite 200 Pro, Switzerland).

### Flow cytometry detection of spleen cell T cell responses

Spleen cells of mice were isolated and cultured in complete RPMI 1640 medium with recombinant TP0435 protein (10 µg/mL) for 24 h at 37℃. The cells were then stimulated with ionomycin, PMA, and BFA (added to complete RPMI 1640 medium at appropriate concentrations) for 6 h at 37℃. After incubation, cells were centrifuged at 1000 rpm for 5 min, and the supernatant was discarded. The cells were washed twice with 250 µL Staining Buffer and resuspended in 100 µL Staining Buffer containing 1 µg BD Fc Block, incubating for 20 min at 4℃. Following a second wash with Staining Buffer, cells were incubated with anti-CD4 and anti-CD8 monoclonal antibodies at 4℃ in the dark for 30 min. After another wash, the cells were fixed and permeabilized with Fixation/Permeabilization solution for 30 min at 4℃. The cells were then washed three times with 1×BD Perm/Wash Buffer, resuspended in the same buffer, and stained with anti-IFN-γ antibody for 30 min at 4°C in the dark. After a final wash, the cells were resuspended in 300 µL Staining Buffer and analysed by flow cytometry to quantify IFN-γ^+^ CD4^+^ and IFN-γ^+^ CD8^+^ T cells.

### Flow cytometric analysis of CD4^+^ and CD8^+^ central memory T cells

Six weeks post the final immunization, mice were euthanized and spleens were aseptically collected. Single-cell suspensions were prepared by mechanical disruption and filtration through a 200-mesh cell strainer. Red blood cells were lysed using erythrocyte lysis buffer, followed by washing with PBS and cell counting. To minimize non-specific binding, cells were first incubated with anti-mouse CD16/CD32 antibodies to block Fc receptors. Cells were then stained with fluorochrome-conjugated monoclonal antibodies against CD3, CD4, and CD8 to identify T cell subsets, along with CD44 and CD62L to characterize memory T cell populations. Staining was performed at 4℃ for 30 min in the dark, followed by two washes with PBS and resuspension. Data were acquired using a flow cytometer and analysed with FlowJo software.

### Extraction of *T. pallidum* DNA and quantitative real-time PCR

DNAs were extracted from tissues of *T. pallidum*-infected rabbits by using a TIANamp Genomic DNA Kit (Tiangen, Beijing, China) following the manufacturer’s protocol. PCR amplification was performed by using a LightCycle 96 apparatus (Roche, Basel, Switzerland). Quantitative analysis of *T. pallidum* gDNA and rabbit gDNA was achieved by using primers targeting *T. pallidum* DNA Polymerase I (*polA*) gene and rabbit collagenase-1 precursor (*MMP-1*) gene, respectively. The sequences of the primers are given as follows: *polA* Sense: 5'-TACGGTGCAAGTGCTCAGAC-3’, Antisense: 5'- CAGGCACATTGTCGGAGGAA −3’; *MMP-1* Sense: 5'-TTGCTTCTTCACACCAGAATGCTGT-3’, Antisense: 5'-GCGTGATCAGGCACTATGTAGCAAT-3’. The primers for quantitative real-time PCR (qPCR) amplification were provided by Genscript Biotech (Nanjing, China). As directed by the manufacturer, qPCR amplifications were performed in 20 µL Taq Pro Universal SYBR Qpcr Master Mix (Vazyme, Nanjing, China) reaction mixture. To ensure accurate quantification of *T. pallidum* burden, we established standard curves using serial 10-fold dilutions of linearized plasmid DNA containing the target *polA* gene (10⁷ to 10¹ copies). The standard curve demonstrated a strong linear relationship between Ct values and *polA* copy numbers within this dynamic range. In parallel, rabbit genomic DNA was serially diluted (200 ng/µL to 1.56 ng/µL) and subjected to RT-qPCR targeting the rabbit *MMP-1* gene to generate a separate standard curve for host DNA quantification. This allowed us to normalize *polA* copy numbers to the corresponding amount of rabbit DNA in each sample. For each tissue sample, the Ct values of *polA* and *MMP-1* were converted into absolute *polA* copy numbers and host DNA concentrations using their respective standard curves. The final results were expressed as *polA* copies per unit of host DNA (copies/μg). Importantly, a Ct value >35 or the presence of abnormal melting curves were predefined as negative, minimizing the likelihood of background amplification or technical artifacts being misinterpreted as true positive signals. All reported values fell within the validated linear range of the standard curve and met the predefined quality control criteria. The following conditions were used for the PCR of *polA* and *MMP-1*: 95°C for 5 min, 40 cycles of 95°C for 10 sec, 60°C for 30 sec. The following is an analysis of melt-curves: 95°C for 10 sec, 65°C for 60 sec, and 97°C for 1 sec.

### Histopathology

On day 21 post-challenge, skin lesions from each rabbit were excised, and a 6-mm punch biopsy was used to obtain standardized tissue subsamples from each lesion. These cutaneous lesion specimens were fixed, embedded in paraffin, and sectioned for histopathological analysis. Following conventional H&E staining, the tissue sections were examined under a light microscope to observe and assess the degree of inflammatory cell infiltration.

### Statistical analysis

Statistical analysis was performed using GraphPad Prism 8.0 software. Data were presented as means ± SDs. The following tests were used to assess significance: two-tailed unpaired Student’s *t*-test for two comparisons; one-way ANOVA test, and Chi-square Test of Independence for multiple comparisons. For data with a small sample size (*n* = 3), the assumptions required for parametric tests (such as normality and homogeneity of variances) are difficult to reliably assess. Therefore, non-parametric tests (Kruskal–Wallis test followed by Dunn’s multiple comparisons test) were applied for data with a small sample size (*n* = 3). A significance level of * *p* < 0.05, ** *p* < 0.01, and *** *p* < 0.001 is considered to indicate significant differences in the statistical results.

## Results

### Construction and characterization of TP0435 mRNA-LNP vaccine

**Figure 1.**
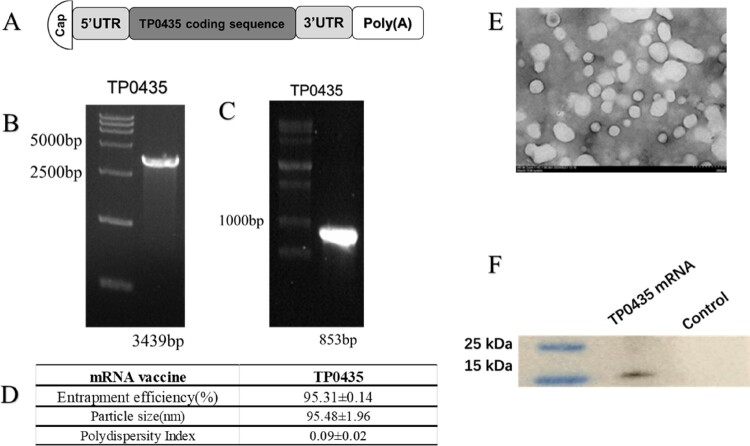
In Vitro Synthesis and Characterization of mRNA-LNP vaccines. (A) The schematic illustration of TP0435 mRNA-LNP vaccine constructs. The mRNA constructs consist of 5’cap followed by 5’UTR, TP0435 mRNA coding sequence, 3’UTR, and poly(A) tail. (B) Detection of the size and integrity of linearized template was assessed by agarose gel electrophoresis. (C) Formaldehyde-denatured agarose gel electrophoresis assessed the integrity and size of mRNA. (D) Physicochemical properties of TP0435 mRNA-LNP vaccine are presented as mean *±* SD. (E) A representative TEM image illustrates the morphology of mRNA-LNP vaccines, with a scale bar of 200 nm. (F) Expression of TP0435 protein was achieved in HEK293 T cells transfected using Lipofectamine3000 for 48 h.

The full sequence of TP0435 protein was selected as the antigenic target for mRNA synthesis. The mRNA constructs consist of a 5’ cap followed by a 5’ UTR, TP0435 coding sequence, a 3’ UTR, and a poly(A) tail (Figure 1A). The full sequence of TP0435 mRNA was provided in Supplementary Table 1. Then it was cloned into pUC57 plasmid. Following plasmid amplification and extraction, linearized template was generated via digestion with the BspQI restriction enzyme (Figure 1B). High-purity mRNA for vaccine purposes was obtained using in vitro transcription from linearized DNA (Figure 1C). TP0435 mRNA was encapsulated in LNPs, with an average size of 95.48 nm, and the polymer dispersity index (PDI) value of LNPs showed good stability of the LNP dispersion system (Figure 1D). The encapsulation efficiency of mRNA-LNP vaccine was higher than 90%. Transmission Electron Microscope (TEM) showed spherical nanoparticles, and no obvious particle aggregation was observed (Figure 1E). Western blot analysis confirmed the expression of a 17 kDa fragment, indicating successful translation and expression of TP0435 protein (Figure 1F).

### TP0435 mRNA-LNP vaccine exhibited no obvious acute toxicity

**Figure 2.**
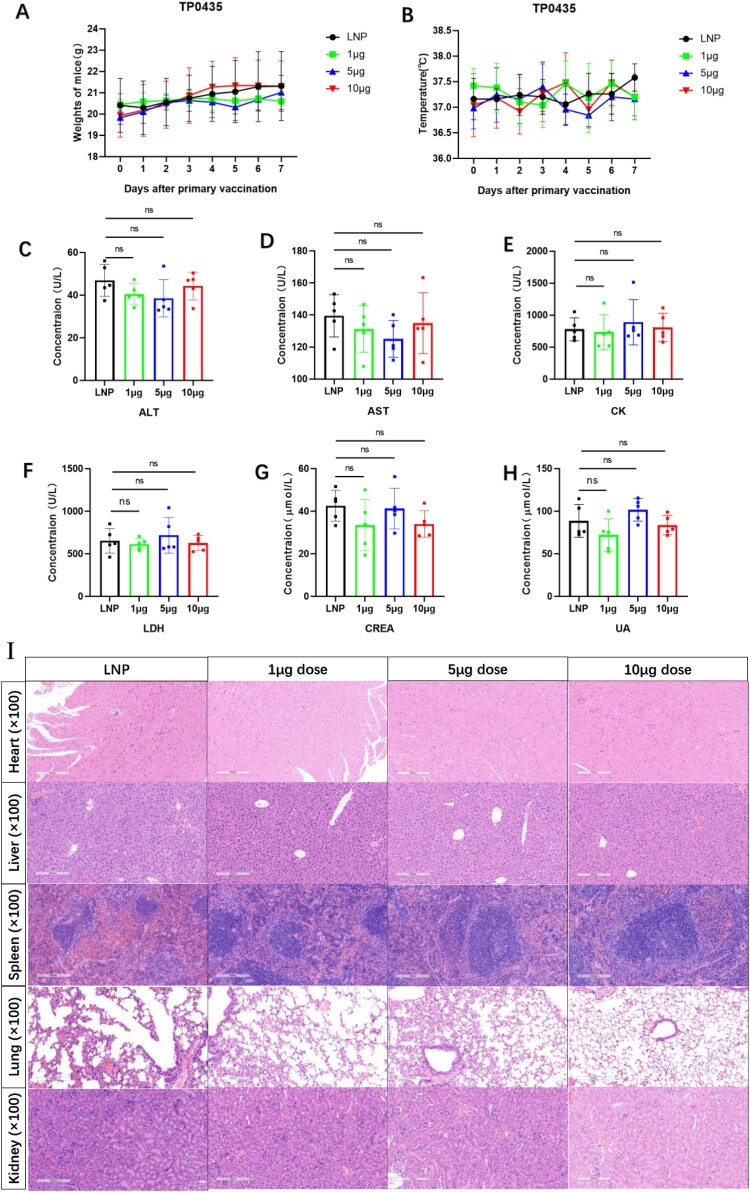
Safety examination of TP0435 mRNA-LNP vaccine. (A) The body weight of immunized mice was monitored for 7 days after the primary immunization. (B) The anal temperature of immunized mice was monitored for 7 days after the primary immunization. (C-H) Assay of serum biochemical indices in immunized mice. ALT, Alanine Aminotransferase. AST, Aspartate Aminotransferase. UA, Uric Acid. CREA, Creatinine. CK, Creatine Kinase. LDH, Lactate Dehydrogenase. (ns, not significant). (I) Histopathological analysis of major organs (Heart, liver, spleen, lung, and kidney tissues) after vaccination. Tissues were collected from mice at 2 weeks after the final immunization and subjected to hematoxylin and eosin (H&E) staining (Scale bar = 200 µm).

To preliminarily evaluate the in vivo toxicity of mRNA vaccine, the body temperature and body weight of immunized mice were monitored for 7 days after the primary immunization. There were no significant differences in body temperature and body weight after vaccination between immunized mice and LNP immunized mice (Figure 2A and B). Based on blood biochemical indicators, we further evaluated the functions of the heart, liver, and kidneys. Among them, creatine kinase (CK) and lactate dehydrogenase (LDH) are indicators of cardiac function; Alanine aminotransferase (ALT) and aspartate aminotransferase (AST) are indicators of liver function; creatinine (CREA) and uric acid (UA) are indicators of renal function. As shown in (Figure 2C–H), there were no significant differences in cardiac function, liver function, and renal function between mRNA-LNP vaccine-immunized mice and LNP-immunized mice, and all data were within the normal range (Figure 2C–H). Moreover, compared with the LNP group, there were no obvious pathological changes in the heart, liver, spleen, lung, and kidney in immunized mice (Figure 2I). Therefore, no obvious acute toxicity was observed within the limited monitoring period.

### TP0435 mRNA-LNP vaccine elicited effective humoral and cellular immune responses in BALB/c mice and NZW rabbits

**Figure 3.**
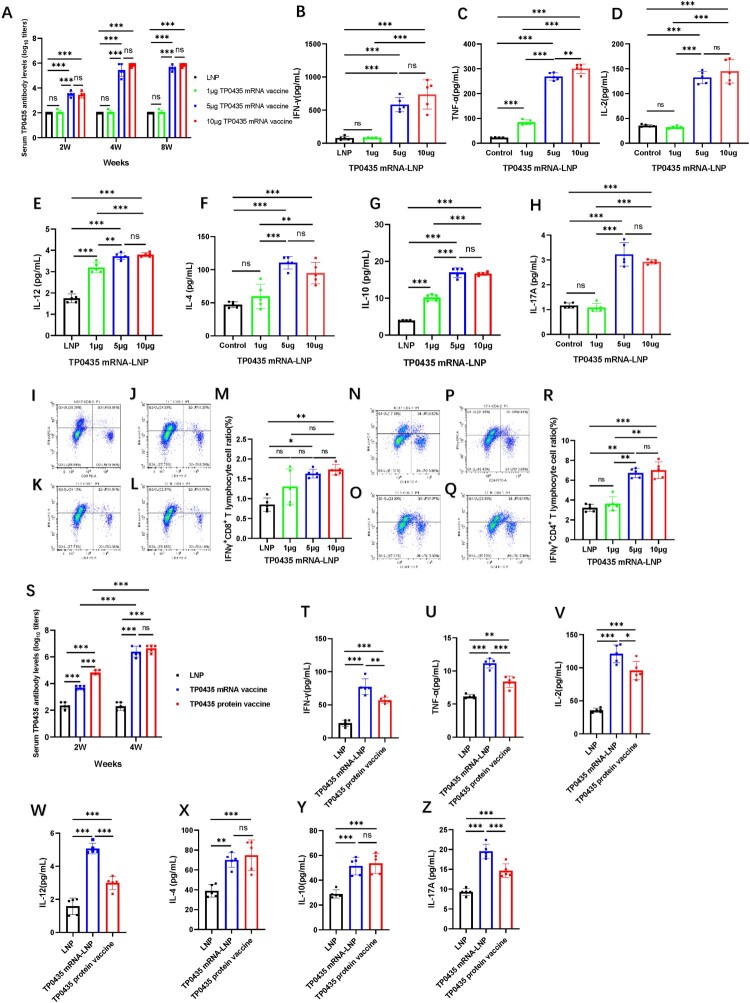
Humoral and cellular immune response of BALB/c mice and NZW rabbits activated by the immunization with TP0435 mRNA-LNP vaccine. (A) IgG antibody levels in mice were determined by indirect ELISA. To determine antibody concentrations (titers), two-fold serial dilutions of serum were made and the endpoint titers were considered to be the last serum dilution with readings higher than the 2.1-fold of the negative controls. (B-H) The levels of IFN-γ, TNF-α, IL-2, IL-12, IL-4, IL-10 and IL-17A were assessed by ELISA in mice splenocytes stimulated with 10 μg recombinant TP0435 protein, with PBS stimulation as blank control. (I-L) The frequencies of IFN-γ^+^ CD8^+^ T cells from mice immunized with 1, 5, and 10 μg dose of TP0435 mRNA vaccine were determined by flow cytometry. (M) Quantification of IFN-γ^+^ CD8^+^ T cells. (N-Q) The frequencies of IFN-γ^+^ CD4^+^ T cells from mice immunized with 1, 5, and 10 μg dose of TP0435 mRNA vaccine were determined by flow cytometry. (R) Quantification of IFN-γ^+^ CD4^+^ T cells. Results are expressed as the means ± SDs. Each set of data is based on measurements derived from five mice. (S) The serum IgG titers against TP0435 in rabbits were determined by indirect ELISA at weeks 2 and 4 post primary immunization. To determine antibody concentrations (titers), two-fold serial dilutions of serum were made and the endpoint titer was considered to be the last serum dilution with readings higher than the 2.1-fold of the negative controls. (T-Z) The levels of IFN-γ, TNF-α, IL-2, IL-12, IL-4, IL-10 and IL-17A by ELISA in splenocytes stimulated with 10 μg recombinant TP0435 protein, with PBS stimulation as blank control. Results are expressed as the means ± SDs. Each set of data is based on measurements derived from five rabbits. (ns, not significant. *P < 0.05, **P < 0.01, ***P < 0.001).

To determine the immune response induced by TP0435 mRNA-LNP vaccine, groups of BALB/c mice were immunized intramuscularly twice with 1, 5 or 10 µg dose of mRNA-LNP vaccines with empty LNP immunization. The interval between every injection was two weeks. The blood samples were collected at week 2, week 4 and week 8. The antigen-specific antibodies were detected by indirect ELISA. As shown in Figure 3(A), 1 µg dose of TP0435 mRNA-LNP vaccine did not induce significant antigen-specific humoral immune responses. After intramuscular injection with 5 µg dose of TP0435 mRNA-LNP vaccine, serum IgG titers significantly increased after the initial immunization and were further elevated after the second immunization. Specifically, at week 2, the highest IgG titers were 1:100 for LNP, 1:200 for 1 µg dose, and 1:6400 for both 5 and 10 µg doses. At week 4, titers for LNP and 1 µg dose remained unchanged at 1:100 and 1:200, respectively, whereas titers for 5 and 10 µg doses increased to 1:819,200. Notably, antibody levels at week 8 remained at similarly high titers, with no significant decline compared to the peak response. Moreover, immunization with 5 µg dose of TP0435 vaccine induced almost the same level of IgG titer as the 10 µg dose. These findings suggested that the 5 µg dose of TP0435 mRNA-LNP vaccine could generate a strong specific antibody response and induce a relatively sustained humoral immune response within the observed timeframe, supporting a certain degree of durability of vaccine-induced immunity. Th1-related cytokines are essential for early clearance of *T. pallidum* in lesions. The clearance of *T. pallidum* primarily depends on Th1-mediated immunity, in which IFN-γ activates macrophages to enhance their phagocytic and bactericidal functions. Therefore, Th1-type cellular immune responses play a critical role in the clearance of T. pallidum. Th2 and Th17 responses play supportive roles in *T. pallidum* infection. In particular, Th17 responses contribute to early host defense by enhancing local inflammatory responses and limiting pathogen dissemination, while Th2 responses are mainly involved in humoral immunity and immune regulation. We examined the levels of IFN-γ, TNF-α, IL-2 and IL-12 produced by mouse splenic cells by ELISA (Figure 3B–E). 5 µg dose of TP0435 mRNA-LNP vaccine could induce dramatically increased levels of IFN-γ, TNF-α, IL-2, and IL-12. To further assess the Th2 and Th17-type cellular immune response induced by the TP0435 mRNA-LNP vaccine, IL-4, IL-10 and IL-17A produced by mouse splenic cells were assessed by ELISA (Figure 3F–H). The results showed that the TP0435 mRNA vaccine could also induce increased levels of IL-4, IL-10, and IL-17A in mice compared with the LNP group, indicating that the vaccine was also capable of eliciting Th2 and Th17-associated responses to a certain extent. Flow cytometry was then used to analyse the proportions of IFN-γ^+^ CD8^+^ and IFN-γ^+^ CD4^+^ cells in splenocytes. The results demonstrated that a 5 μg dose of TP0435 mRNA-LNP was sufficient to induce an increase in the proportion of IFN-γ^+^ CD8^+^ and IFN-γ^+^ CD4^+^ cells (Figure 3I–R). This finding was consistent with the IFN-γ secretion levels detected in the supernatant of spleen cell cultures. Taken together, these findings indicated that the TP0435 mRNA-LNP vaccine predominantly elicited a Th1-biased cellular immune response and 5 µg dose of TP0435 mRNA-LNP vaccine might be the optimal dose to elicit Th1-related immune responses in BALB /c mice.

Based on the results of antigen-specific humoral and T-cell immune responses in BALB/c mice elicited by the TP0435 mRNA-LNP vaccine. 5 µg dose of TP0435 mRNA-LNP vaccine was used for further immunization in rabbits. The sequence and expression of the TP0435 recombinant protein are presented in the Supplementary Table 2 and Supplementary Figure 1. Each rabbit was immunized once every two weeks for a total of two times. Blood was collected on day 14 and day 28. As shown in Figure 3(S), the TP0435 mRNA-LNP vaccine induced higher endpoint titers of specific antibodies starting at week 2 compared with LNP-immunized rabbits but lower titers than those induced by the TP0435 protein vaccine. After the primary immunization, the maximum endpoint titer in the TP0435 mRNA-LNP group reached 1:6,400, whereas the TP0435 protein vaccine group reached 1:204,800. Following the booster immunization, antibody titers were markedly increased in both groups, reaching a maximum endpoint titer of 1:6,553,600, with no significant difference between the TP0435 mRNA-LNP and protein vaccine groups. Both groups exhibited significantly higher titers after the second immunization compared with the primary immunization. These results indicated that TP0435 mRNA-LNP vaccine was able to induce strong antigen-specific humoral responses. IFN-γ, TNF-α, IL-2, IL-12, IL-4, IL-10, and IL-17A produced by rabbit splenic cells were measured by ELISA (Figure 3T–Z). Compared with rabbits immunized with LNP alone, the IFN-γ, TNF-α, IL-2, IL-12, and IL-17A levels were significantly elevated in both the mRNA and protein vaccine groups, with higher levels observed in the mRNA vaccine group. In addition, the TP0435 mRNA-LNP and protein vaccine also induced higher levels of Th2-associated cytokines (IL-4 and IL-10) compared with the LNP group, whereas no significant difference was observed between the two groups. The results indicated that TP0435 mRNA-LNP vaccine could simultaneously induce Th1, Th2, and Th17-associated responses, with a predominant Th1 bias in NZW rabbits.

### TP0435 mRNA-LNP vaccine induced CD4^+^ central memory T cell response

**Figure 4.**
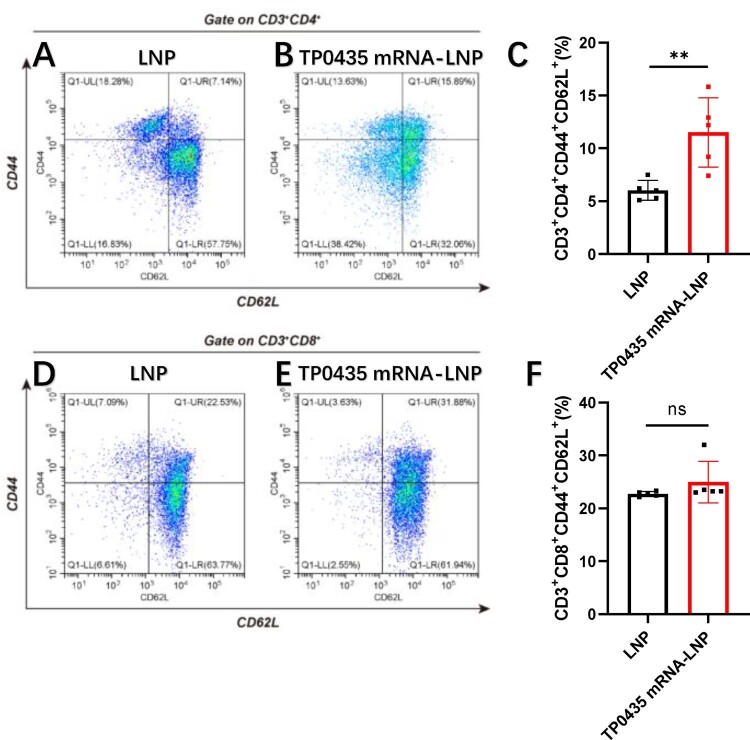
Changes in central memory T cell (Tcm) levels in the spleen of BALB/c mice at 6 weeks after immunization with the TP0435 mRNA vaccine. (A) Frequency of CD4^+^ Tcm cells in the LNP group. (B) Frequency of CD4^+^ Tcm cells in the TP0435 mRNA-LNP vaccine group. (C) Quantitative analysis of CD4^+^ Tcm cell frequency. (D) Frequency of CD8^+^ Tcm cells in the LNP group. (E) Frequency of CD8^+^ Tcm cells in the TP0435 mRNA-LNP vaccine group. (F) Quantitative analysis of CD8^+^ Tcm cell frequency.

Central memory T cell (Tcm) is a key indicator for evaluating long-term protective immunity. Tcm possess high proliferative capacity and the ability to rapidly differentiate into effector T cells upon antigen re-exposure, thereby mediating rapid and effective immune responses. Since protective immunity against *T. pallidum* is primarily dependent on CD4^+^ T cell–mediated Th1 responses, the assessment of CD4^+^ Tcm is particularly important for determining the durability of vaccine-induced immunity. Meanwhile, CD8^+^ Tcm serves as a reservoir of cytotoxic T cells that can differentiate into effector cells upon re-exposure and contribute to the elimination of infected cells, and their evaluation helps determine whether the vaccine elicits a comprehensive cellular immune response. Based on the overall evaluation of humoral and cellular immune responses induced by different doses of the TP0435 mRNA vaccine, the 5 μg dose was selected for subsequent experiments. At 6 weeks after the final immunization, splenocytes were analysed by flow cytometry. The results showed that the frequency of CD4^+^ Tcm was significantly increased in the TP0435 mRNA-LNP vaccine group compared with the LNP group (Figure 4A–C), whereas no significant difference was observed in CD8^+^ Tcm (Figure 4D–F). These findings indicated that the TP0435 mRNA-LNP vaccine preferentially induces a CD4^+^ T cell–mediated central memory response and has the potential to confer long-term immunological protection.

### Syphilis serological analysis of NZW rabbit sera

Considering that TP0435 is a major antigen used in syphilis serological assays, sera from immunized NZW rabbits were evaluated by TPPA, GICA, and RPR to determine whether TP0435-specific antibodies induced by the mRNA-LNP and protein vaccines interfere with serological detection. As shown in the Supplementary Table 3, 2 weeks after primary immunization, TPPA, GICA, and RPR were all negative in the LNP and TP0435 mRNA vaccine groups. In contrast, the TP0435 protein vaccine group showed GICA positivity, TPPA borderline reactivity, and negative RPR. 2 weeks after booster immunization, the LNP group remained negative, whereas both the TP0435 mRNA and protein vaccine groups exhibited GICA positivity and TPPA borderline reactivity, with RPR remaining negative in all groups.

### TP0435 mRNA-LNP vaccine immunization attenuated lesion development

**Figure 5.**
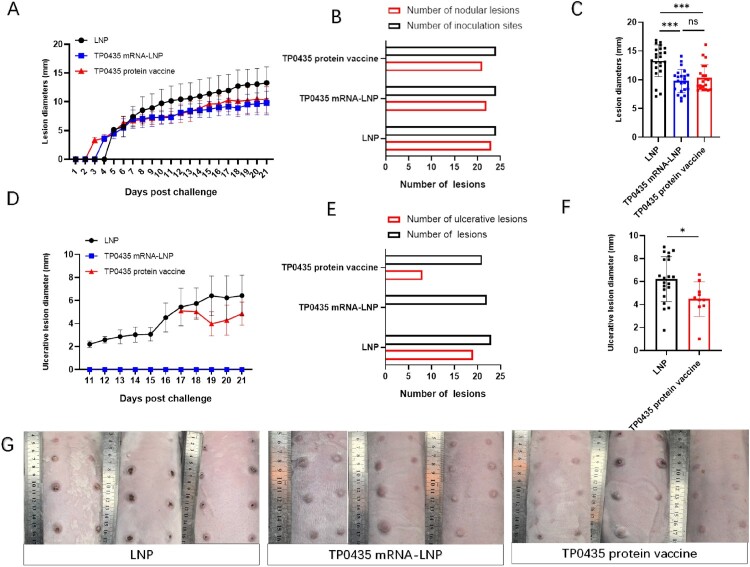
Immunization with TP0435 mRNA-LNP vaccine could attenuated lesion development. (A) The diameter of each induration was measured daily over 21 days post-intradermal challenge with *T. pallidum*. (B) The number of total indurations on day 21 post-intradermal challenge with *T. pallidum*. (C) The diameter of each induration on day 21 post-intradermal challenge with *T. pallidum*. (D) The diameter of each ulceration was measured daily over 21 days post-intradermal challenge with *T. pallidum*. (E) The number of total ulcerations on day 21 post-intradermal challenge with *T. pallidum*.(F) The diameter of each ulceration on day 21 post-intradermal challenge with *T. pallidum*. Results are expressed as the means ± SDs. (ns, not significant. *P < 0.05, **P < 0.01, ***P < 0.001). (G) Lesions on the backs of the rabbits on day 21 post-intradermal challenge with *T. pallidum*.

Two weeks post the booster immunization, each rabbit was challenged intradermally with 0.1 ml of 1 × 10^6^ freshly isolated *T. pallidum* Nichols strain in 0.9% saline at each of 8 sites. During the subsequent 21 days, induration and ulceration were monitored daily for diameters. As shown in Figure 5(A), obvious indurations appeared in rabbits on day 5, 4, and 3 in the LNP group, TP0435 mRNA-LNP vaccine group, and TP0435 protein vaccine group, respectively. Lesion development was delayed in both the TP0435 mRNA-LNP vaccine and TP0435 protein vaccine groups compared with the LNP group. On day 21, indurations were present in all rabbits, with a total of 23, 22, and 21 lesions observed in the LNP group, TP0435 mRNA-LNP vaccine group, and TP0435 protein vaccine group, respectively (Figure 5B). On day 21 post-challenge, the diameters of nodular lesions were also markedly reduced in vaccinated groups, with mean diameters of 13.30 ± 2.76 mm in the LNP group, 9.78 ± 2.07 mm in the TP0435 mRNA-LNP vaccine group, and 10.33 ± 2.28 mm in the TP0435 protein vaccine group, with no significant difference between the mRNA and protein vaccine groups (Figure 5C). Moreover, ulcerations appeared on day 11 in the LNP group and day 17 in the TP0435 protein vaccine group, whereas no ulceration was observed in the TP0435 mRNA-LNP vaccine group by day 21 (Figure 5D). As presented in Figure 5(E), the number of ulcerations was significantly lower in the TP0435 protein vaccine group (8/21) than in the LNP group (19/23). In addition, the diameters of ulcerations on day 21 were also markedly smaller in vaccinated rabbits. Specifically, the mean ulcer diameter in the LNP group was 6.43 ± 1.76 mm, whereas no ulcer formation was observed in the mRNA vaccine group, and a reduced diameter of 4.86 ± 1.01 mm was observed in the protein vaccine group (Figure 5F). All skin lesions are shown in Figure 5(G). Collectively, these results indicated that immunization with the TP0435 mRNA-LNP vaccine significantly delayed lesion development and effectively prevented ulcer formation. Therefore, the TP0435 mRNA-LNP vaccine exhibited higher efficacy in delaying induration development, with an overall superior protective effect against ulceration.

### TP0435 mRNA-LNP vaccine immunization inhibited *T. pallidum* dissemination

**Figure 6.**
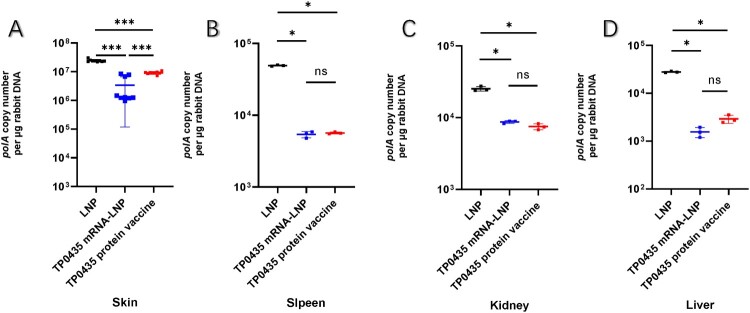
Immunization with TP0435 inhibited *T. pallidum* dissemination. The *T. pallidum* burden was evaluated in rabbits immunized with LNP (*N* = 3), TP0435 mRNA-LNP vaccine (*N* = 3) and TP0435 protein vaccine (*N* = 3) using quantitative real-time PCR to measure the *polA* DNA concentrations in the lesion biopsies (A) and distal organs including spleen (B), kidney (C) and liver (D). Results of each tissue type were normalized according to the concentration of rabbit gDNA. One-way ANOVA test was used to analyse the results. Repeatability analysis was performed on three tissue samples of each rabbit organ. Each dot represents the mean *polA* DNA concentration of three individually selective tissue samples from every organ of each rabbit. Horizontal lines represent mean values (ns, not significant. *P < 0.05, **P < 0.01, ***P < 0.001).

To evaluate the ability of mRNA-LNP vaccines immunization to prevent *T. pallidum* dissemination, all rabbits were euthanized on day 21 post-challenge. The total DNA was extracted from nine cutaneous lesions in each group at the initial infection sites as well as distal tissues or organs and qPCR was performed to determine *T. pallidum* DNA concentration to assess the treponemal burdens. As shown in Figure 6(A), the results revealed that the *T. pallidum* load was significantly decreased at cutaneous lesions in rabbits immunized with TP0435 mRNA-LNP vaccine and TP0435 protein vaccine compared with LNP group, with the mRNA vaccine group showing lower *T. pallidum* burden than the protein vaccine group. Notably, the *T. pallidum* loads in the LNP group were approximately 7-fold higher than those in the mRNA vaccine group and 2.7-fold higher than those in the protein vaccine group. The *T. pallidum* burdens in the distal organs of the animals were also assessed. After immunization with TP0435 mRNA-LNP vaccine and TP0435 protein vaccine, the burdens in the liver, spleen, and kidney similarly decreased compared with the LNP group (Figure 6B–D). These results indicated that both the mRNA vaccine and the protein vaccine could effectively reduce the *T. pallidum* loads in skin lesions and distal organs, with the mRNA vaccine group showing a lower *T. pallidum* load in skin lesions.

### The TP0435 mRNA-LNP vaccine immunization promoted inflammatory infiltration

**Figure 7.**
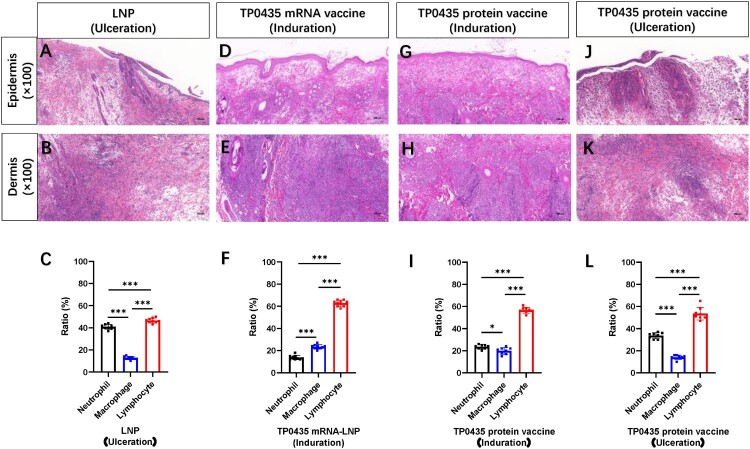
Histopathological analysis of primary cutaneous lesions on 21 days post-intradermal challenge with *T. pallidum*. On day 21 post *T. pallidum* challenge, cutaneous tissues from LNP group (A, B), TP0435 mRNA-LNP vaccine group (D, E) and TP0435 protein vaccine (G, H, J, K) were sliced and stained with H&E (Scale bar = 100 µm). Semi-quantitative analysis of inflammatory cell infiltration, assessing neutrophils, macrophages, and lymphocytes were performed in the skin lesions of rabbits immunized with LNP (C), TP0435 mRNA-LNP vaccine (F) and TP0435 protein vaccine (I, L).

On day 21 post-challenge, H&E-stained sections from primary cutaneous lesion sites of rabbits were performed to evaluate the intensities of inflammatory infiltration and cell types. In the LNP group, H&E sections revealed pronounced disruption of epidermal and dermal architecture, accompanied by infiltration of neutrophils, macrophages, and lymphocytes (Figure 7A and B). Semi-quantitative analysis showed that neutrophils accounted for 40.78% of infiltrating cells at ulcerative control challenge sites, whereas macrophages and lymphocytes represented 12.67% and 52.22%, respectively, indicating a neutrophil-dominant inflammatory response (Figure 7C). In contrast, tissues from the TP0435 mRNA-LNP group exhibited largely preserved epidermal and dermal structures (Figure 7D and E). At the challenge sites presenting as nodules in the TP0435 mRNA-LNP group, neutrophils constituted 13.78% of inflammatory cells, while macrophages and lymphocytes increased to 23.44% and 62.78%, respectively (Figure 7F). In the TP0435 protein-vaccinated group, histopathological features differed between lesion types. Nodular lesions displayed largely preserved epidermal and dermal architecture (Figure 7G and H), with inflammatory infiltrates composed of 23.50% of neutrophils, 19.88% of macrophages, and 56.63% of lymphocytes (Figure 7I). In contrast, ulcerative lesions showed disruption of epidermal and dermal structures similar to that observed in the LNP group (Figure 7J and K), accompanied by inflammatory infiltrates in which neutrophils, macrophages, and lymphocytes accounted for 33.63%, 14.13%, and 53.50%, respectively (Figure 7L). Overall, these findings indicated that vaccination with TP0435 mRNA or protein markedly reduced neutrophil infiltration and increased macrophage and lymphocyte presence in nodular lesions, whereas ulcerative lesions in the TP0435 protein group showed slight neutrophil reduction and increases in macrophage and lymphocyte infiltration.

## Discussion

*T. pallidum* surface exposed antigens are regarded as the most promising vaccine candidates. The identification and characterization of outer membrane proteins (OMPs) have been challenging due to the fragility of OMPs and the difficulty of genetically manipulating and cultivating them in vitro until recently [22]. Over the past few years, advances in computational methods for predicting OMPs of *T. pallidum*, coupled with the application of functional and biophysical assays to validate these predictions, have led to the identification of several vaccine candidates [23, 24]. OMPs can be classified in three main groups according to their putative functions, which include adhesion function, transport function and other functions [25]. Among these putative OMPs, adhesion to host cells and the extracellular matrix (ECM) is critical for the establishment of infection and subsequent dissemination of *T. pallidum*. mRNA vaccine of *T. pallidum* has not been reported so far. TP0435 is a lipoprotein with adhesin function, encompassing an eight-stranded anti-parallel b-barrel with a basin-like domain located at one end [26]. TP0435 might be localized between the bacterial surface and the periplasmic space [15]. Such evidence led us to hypothesize that TP0435 could also be a possible vaccine candidate. Furthermore, Parveen et al. [15] found the immunization of rabbits with a Tp0435-expressing *B. burgdorferi* strain induced humoral responses and splenocyte proliferation, but failed to protect from *T. pallidum* infection. In this study, we investigated whether immunization with the adhesion TP0435 mRNA-LNP vaccine was able to induce a strong and specific immune response and offer protection against T. pallidum infection. The results provided evidence that TP0435 mRNA-LNP vaccines elicited antigen-specific humoral and T-cell immune responses both in mice and rabbits. Furthermore, in contrast to the previous study, compared to rabbits immunized with LNP, rabbits immunized with TP0435 mRNA-LNP vaccine showed attenuated lesion development and inhibition of treponemal dissemination to distant organs. The strategy failed to confer protective immunity against the *T. pallidum* challenge, which might be attributed to several reasons. TP0435 exhibited limited and stochastic surface exposure within the carrier, and the rapid clearance of the bacteria restricted antigen availability, thereby limiting the induction of fully functional antibodies and Th1-type cellular responses. Moreover, the study did not employ adjuvants or assess Th1/Th2 cytokine profiles, such as IFN-γ or IL-12, preventing evaluation of whether the cellular response was sufficient to mediate opsonophagocytic clearance of treponemes. In contrast, our TP0435 mRNA-LNP and protein vaccines (formulated with adjuvant) elicited potent humoral and Th1-biased cellular immunity. These findings indicated that while this carrier-based approach might be less effective than conventional protein-adjuvant formulations or contemporary mRNA-LNP platforms, TP0435 remains a promising target for syphilis vaccine development and warrants inclusion in future multi-antigen or next-generation vaccine designs.

Dose selection in this study was guided by established nonclinical vaccination practices and by the intrinsic differences between protein and mRNA-based vaccine platforms. The 5 µg dose was initially selected based on the mouse immunogenicity experiment, in which it elicited strong antigen-specific humoral and cellular immune responses without observable toxicity. Importantly, mRNA vaccines rely on in vivo antigen expression rather than direct antigen mass delivery. Therefore, effective immune priming can be achieved at low microgram doses, and dose equivalence between protein and mRNA platforms is not based on mass equivalence. In preclinical studies of SARS-CoV-2 mRNA vaccines, robust immune responses were typically induced in animal models at doses ranging from 1 to 10 µg. Furthermore, clinically licensed COVID-19 mRNA vaccines, such as BNT162b2 and mRNA-1273, are administered at fixed microgram doses (30 and 100 µg per dose in adults, respectively) rather than using strict body-weight-based scaling [27, 28]. These examples support the general principle that mRNA vaccine dosing is platform-dependent and not linearly extrapolated according to body mass. Nevertheless, we acknowledge that a formal dose-escalation or dose–response study was not performed in rabbits in this work. The selected 5 µg dose should therefore be considered a preliminary, feasibility-based choice for proof-of-concept evaluation rather than an optimized protective dose. It remained possible that alternative dosing strategies could further enhance protective efficacy.

The TP0136 mRNA-LNP vaccine reported recently was designed based on selected T-cell epitopes, whereas our TP0435 vaccine is a full-length mRNA construct encoding the entire TP0435 protein. This fundamental difference in antigen design likely contributes to the distinct immune response patterns and protective outcomes observed between the two studies. In the mRNA vaccine research targeting TP0136, the protein vaccine induced a stronger Th1-type cellular immune response compared with the TP0136 mRNA vaccine. Correspondingly, the TP0136 protein vaccine demonstrated better protection against ulcer formation than its mRNA counterpart. This result suggested that, in the context of a T-cell epitope-based antigen, the magnitude of Th1 cellular immunity may be closely associated with ulcer protection. In contrast, in our study using the full-length TP0435 antigen, we observed that the TP0435 mRNA-LNP vaccine induced a stronger Th1-type cellular immune response than the TP0435 protein vaccine. Consistently, the TP0435 mRNA-LNP vaccine conferred superior protection against ulcer formation compared with the protein vaccine. Taken together, the two studies collectively suggested that the level of Th1-type cellular immunity was closely associated with the control of lesion progression and ulcer development.

TP0435 is a well-recognized antigenic component of *T. pallidum* and has been used in certain serological assays for syphilis diagnosis. TP0435 antibodies induced by the TP0435 mRNA vaccine might not be fully distinguishable from those generated by *T. pallidum* infection using conventional serological tests. Our further experiments showed that after TP0435 mRNA vaccination, serological tests were partially positive (TPPA was equivocal, colloidal gold assay was positive, and RPR remained negative). However, sera from infected individuals are generally positive in multiple serological assays, including TPPA, colloidal gold assays, and RPR tests. The results provided a reference point for differentiating vaccine-induced antibodies from infection-induced antibodies. Moreover, it is important to note that natural syphilis infection induces antibody responses against a broad range of *T. pallidum* antigens rather than a single protein. In addition to TpN17 (TP0435) and TpN47 (TP0574), several other proteins including TpN15 (TP0171), Tp0326 (Tp92), Tp0453, TpF1 (TP1038), Tp0965, Tp0100, Tp1016, as well as flagellar proteins FlaB1 (TP0868), FlaB2 (TP0792), FlaB3 (TP0870), and Tp0463, have been reported to react with patient sera and show high seroreactivity in ELISA-based assays [29–33]. Therefore, infection elicits a multi-antigen antibody profile, whereas TP0435 mRNA vaccination primarily induces antibodies against TP0435, providing an important basis for distinguishing vaccine-induced from infection-induced antibodies. Furthermore, in human use, pre-vaccination serological screening for syphilis would typically be performed to confirm recipients are not infected, further minimizing potential confounding.

An effective syphilis vaccine should prevent chancre development and treponemal transmission. In this study, the TP0435 mRNA-LNP vaccine immunized rabbits showed a significant delayed onset time of induration and a smaller lesion diameter. Notably, the TP0435 mRNA-LNP vaccine provided superior clinical protection, completely preventing ulcer formation. The ability of a vaccine to prevent ulcer formation holds broader implications for sexually transmitted infection (STI) prevention. Syphilitic ulcers cause mucosal damage, which creates entry points that facilitate the transmission of other pathogens. Therefore, the TP0435 mRNA-LNP vaccine could preserve mucosal barriers and thereby reduce the risk of infection from other STIs. Consistent with these findings, qPCR analysis further revealed significantly lower *T. pallidum* burdens at the primary lesion site and distal organs in rabbits immunized with TP0435 mRNA-LNP vaccine. Based on the above results, although TP0435 immunization did not induce complete protection, it partially prevented chancre development and treponemal transmission.

In addition to macroscopic observations, we performed detailed histopathological analyses of the challenge sites to better characterize local tissue responses. Based on H&E-stained sections, we conducted a semi-quantitative assessment of inflammatory cell infiltration at lesion sites, focusing on neutrophils, macrophages, and lymphocytes. In the LNP group, H&E sections revealed pronounced disruption of epidermal and dermal architecture accompanied by abundant neutrophil infiltration, together with macrophages and lymphocytes. Semi-quantitative analysis confirmed that neutrophils constituted the predominant inflammatory cell population, consistent with an uncontrolled, neutrophil-dominated inflammatory response. This type of inflammation is well recognized to contribute to tissue destruction, ulceration, crust formation, and purulent exudate during *T. pallidum* infection in the absence of effective antigen-specific adaptive immunity [34]. In contrast, tissues from the TP0435 mRNA-vaccinated rabbits exhibited largely preserved epidermal and dermal structures, with markedly reduced or absent ulceration and purulent exudate. Semi-quantitative analysis demonstrated a clear shift in inflammatory cell composition, characterized by increased infiltration of macrophages and lymphocytes and a relative reduction in neutrophils. This altered cellular profile is indicative of a vaccine-induced, Th1-biased cellular immune response, in which activated macrophages and antigen-specific lymphocytes play a central role in controlling *T. pallidum* infection while limiting excessive tissue damage. These histopathological and semi-quantitative findings provided mechanistic support for the observed differences in lesion severity between groups. Importantly, they indicate that vaccine-mediated protection is reflected not only in macroscopic outcomes but also in the quality and composition of the local immune response at the tissue level.

During syphilis infection, the clearance of *T. pallidum* is primarily mediated by cell-mediated immunity, with infiltration of T cells and macrophages closely associated with the resolution of primary and secondary lesions [35–37]. Primary lesions are predominantly composed of CD4^+^ T cells, macrophages, and natural killer (NK) cells, whereas secondary lesions exhibit a relatively higher proportion of CD8^+^ T cells [38, 39]. A strong delayed-type hypersensitivity (DTH) response is critical for effective pathogen clearance, which requires local production of Th1-type cytokines, including IFN-γ, IL-2, and IL-12. These cytokines are primarily secreted by CD4^+^ T cells, with additional contributions from NK cells, CD8^+^ T cells, and dendritic cells [40–43]. Th1-mediated activation of macrophages promotes opsonophagocytosis of *T. pallidum*, which is considered the major mechanism of clearance [44–47]. Humoral immunity acts in concert with cellular immunity, as opsonophagocytosis depends on immune serum, while neither passive transfer of antibodies nor adoptive transfer of T cells alone confers sterilizing immunity [48–50]. Although the TP0435 mRNA vaccine induced a robust humoral response, high antibody titers did not necessarily equate to sterilizing immunity in *T. pallidum* infection. In the present study, we primarily quantified IgG levels but did not directly evaluate functional antibody properties such as neutralizing or opsonophagocytic activity. Therefore, while the data clearly demonstrated strong immunogenicity, the qualitative functional capacity of these antibodies remained to be fully characterized. Given the biology of *T. pallidum*, protection is widely considered to depend heavily on Th1-mediated cellular immunity, particularly on IFN-γ-driven macrophage activation that enhances bacterial clearance. Consistently, our vaccine elicited a pronounced Th1-biased response, including increased IFN-γ, IL-2, and TNF-α production, and elevated frequencies of IFN-γ⁺ CD4⁺ and CD8⁺ T cells, as well as higher levels of macrophages and lymphocytes infiltration suggesting that the observed delay in lesion development and reduction in bacterial burden may be more closely associated with cellular immune mechanisms rather than solely antibody-mediated effects.

In this study, we first identified that TP0435 might be a potential vaccine candidate for syphilis. Furthermore, this is the first study that explored the immunogenicity and immune protection of TP0435 mRNA-LNP vaccine and confirmed the feasibility and effectiveness of this mRNA-LNP vaccine in rabbit model of syphilis. Unlike traditional protein vaccines, our mRNA-LNP vaccine showed robust immune responses without additional adjuvants, which could not be used in humans due to the side effects [51]. It is imperative to note that several limitations existed in this study. Firstly, although BALB/c mice have well-characterized immune systems, are readily available, and are widely used in vaccine research, they allow reproducible and comparable assessment of antigen-specific humoral and cellular responses. This strain is Th2-biased, which may affect the detectable levels of Th1-type cytokines and potentially underestimate the cellular immune responses required for *T. pallidum* clearance. Therefore, Th2 bias should be taken into consideration when interpreting Th1-type readouts. Future studies may consider using additional mice strains to more accurately evaluate Th1-type immune responses. Besides, the primary objective of this study was to evaluate the antigen-specific immune response induced by TP0435 mRNA delivered via LNP. For this reason, we included an empty LNP group to distinguish immune responses resulting from TP0435 antigen expression from those potentially caused by the delivery vehicle itself. However, the absence of a blank control group, a PBS control group, a saline-injected control group, and TiterMax Gold adjuvant control groups limited the ability to fully characterize the baseline immune status and to quantify the magnitude of non-specific immune activation induced by LNP or TiterMax Gold adjuvant relative to physiological background levels. In terms of safety, a more comprehensive safety assessment, including monitoring after booster immunization, dynamic serum biochemical analysis during the acute phase, and longer-term observation, will be necessary in future studies to fully characterize the safety profile of the mRNA vaccine. Besides, this study examined the immune protection only four weeks after the primary immunization and observed it only three weeks after the *T. pallidum* challenge, lacking further long-term observation. Long-term protection and observation in future studies through delayed challenge experiments should be evaluated. Although the mRNA vaccine induced a stronger Th1-type cellular immune response, the precise mechanism underlying its superior protection against ulcer formation remained unclear. Therefore, further experiments are required to elucidate this mechanism. Regarding strain variability, our current experiments were conducted using the *T. pallidum* Nichols strain, which remains the standard laboratory strain for controlled rabbit challenge models. However, the other strains such as MexicoA and SS14/Sea81–4 strains were not included in this study and should be investigated in the future work. In addition, the number of animals used in this study was constrained by institutional ethical requirements and the principles of the 3Rs (Replacement, Reduction, and Refinement) governing animal experimentation. In particular, the principle of Reduction requires that the minimum number of animals necessary to achieve scientific objectives be used, provided that meaningful and interpretable data can still be obtained. The rabbit syphilis challenge model involves significant ethical considerations, specialized housing conditions, and relatively high animal welfare burden. Therefore, the study was designed as an initial proof-of-concept experiment using the minimum number of animals permitted under ethical approval. However, the relatively small number of animals used in the *T. pallidum* challenge experiments resulted in low statistical power, which may affect the robustness and generalizability of the results. With respect to model standardization, the rabbit challenge procedures were conducted according to previously published and widely used protocols. We acknowledge, however, that minor procedural variability such as residual dorsal hair at some inoculation sites might influence local lesion development. More stringent and uniform preparation of inoculation sites, including more thorough hair removal, would further enhance reproducibility. In addition, a more standardized sampling strategy in future experiments such as fixed depth sampling from the epidermis, distinguishing between the central and peripheral regions of the chancre, and differentiating ulcerative from non-ulcerative lesions, should be adopted. These improvements will help ensure greater consistency and interpretability of histopathological data in future studies. Moreover, systematic dose-escalation studies in mice and rabbits to evaluate potential immune over-activation and to define the optimal dose would be valuable for future translational development.

## Conclusion

In brief, our study results suggested that TP0435 represented a highly promising vaccine candidate. Immunization with TP0435 mRNA-LNP vaccine could induce antigen-specific humoral and T-cell immune responses. Moreover, rabbits immunized with TP0435 mRNA-LNP vaccine showed strong efficiency in attenuating lesion development and provided complete protection from ulceration. However, a single immunogen of the mRNA-LNP vaccine could not provide complete protection. Future studies should consider the development of a multicomponent vaccine containing more than two protective mRNA-LNP vaccines. The challenges in syphilis vaccine research include the difficulty of culturing *T. pallidum* in vitro, the inability to perform gene editing on this pathogen, the scarcity and fragility of its outer membrane proteins. With the continuous advancement of technologies, there have been several studies reporting that *T. pallidum* could be cultured in vitro and genetic manipulation of this pathogen is achievable, providing new insights for syphilis vaccines research.

## Supplementary Material

Supplemental Material

## Data Availability

The original data of this article will be provided by the authors. None.
