# Supplementary material for: An mRNA-LNP vaccine expressing TP0435 provides protective immunity in rabbits against Treponema pallidum challenge
Source: Emerg Microbes Infect. 2026 Jun 21;15(1):2685927. doi: 10.1080/22221751.2026.2685927 (PMC13288832; doi:10.1080/22221751.2026.2685927)
Supplement: Supplemental Material [file TEMI_A_2685927_SM9072.docx]

Supplementary table 1. The sequence of TP0435 mRNA.

| 5’UTR |
| --- |
| UAAUACGACUCACUAUAAGGAGAAUAAACUAGUAUUCUUCUGGUCCCCACAGACUCAGAGAGAACCCGCCACC |
| TP0435 CDS |
| AUGAAAGGAUCUGUCCGCGCGCUGUGCGCGUUCCUGGGUGUUGGAGCGCUCGGUAGCGCUUUGUGUGUCUCGUGCACAACCGUGUGUCCGCACGCCGGGAAGGCCAAAGCGGAAAAGGUAGAGUGCGCGUUGAAGGGAGGUAUCUUUCGGGGUACGCUACCUGCGGCCGAUUGCCCGGGAAUCGAUACGACUGUGACGUUCAACGCGGAUGGCACUGCGCAAAAGGUAGAGCUUGCCCUUGAGAAGAAGUCGGCACCUUCUCCUCUUACGUAUCGCGGUACGUGGAUGGUACGUGAAGACGGAAUUGUCGAACUCUCGCUUGUGUCCUCGGAGCAAUCGAAGGCACCGCACGAGAAAGAGCUGUACGAGCUGAUAGACAGUAACUCCGUUCGCUACAUGGGCGCUCCCGGCGCAGGAAAGCCUUCAAAGGAGAUGGCGCCGUUUUACGUGCUGAAAAAAACAAAGAAAUAG |
| 3’UTR |
| UGACUCGAGCUGGUACUGCAUGCACGCAAUGCUAGCUGCCCCUUUCCCGUCCUGGGUACCCCGAGUCUCCCCCGACCUCGGGUCCCAGGUAUGCUCCCACCUCCACCUGCCCCACUCACCACCUCUGCUAGUUCCAGACACCUCCCAAGCACGCAGCAAUGCAGCUCAAAACGCUUAGCCUAGCCACACCCCCACGGGAAACAGCAGUGAUUAACCUUUAGCAAUAAACGAAAGUUUAACUAAGCUAUACUAACCCCAGGGUUGGUCAAUUUCGUGCCAGCCACACCCUGGAGCUAGC |
| Poly(A) Tail |
| AAAAAAAAAAA |

Supplementary table 2. The sequence of TP0435 recombinant protein.

| MCVSCTTVCPHAGKAKAEKVECALKGGIFRGTLPAADCPGIDTTVTFNADGTAQKVELALEKKSAPSPLTYRGTWMVREDGIVELSLVSSEQSKAPHEKELYELIDSNSVRYMGAPGAGKPSKEMAPFYVLKKTKKLEHHHHHH* |
| --- |

Supplementary table 3. Serological detection of syphilis in New Zealand rabbits 2 weeks after primary and booster immunization with TP0435 mRNA vaccine and protein vaccine.

Table legend: Sera were collected from New Zealand rabbits 2 weeks after primary and booster immunization with TP0435 mRNA vaccine and protein vaccine, and subjected to syphilis serological testing using the Treponema pallidum Particle Agglutination assay (TPPA), Gold Immunochromatographic Assay (GICA), and Rapid Plasma Reagin test (RPR).

Supplementary figure 1. Expression, optimization, solubility, purification and dialysis analysis of the TP0435 recombinant protein.


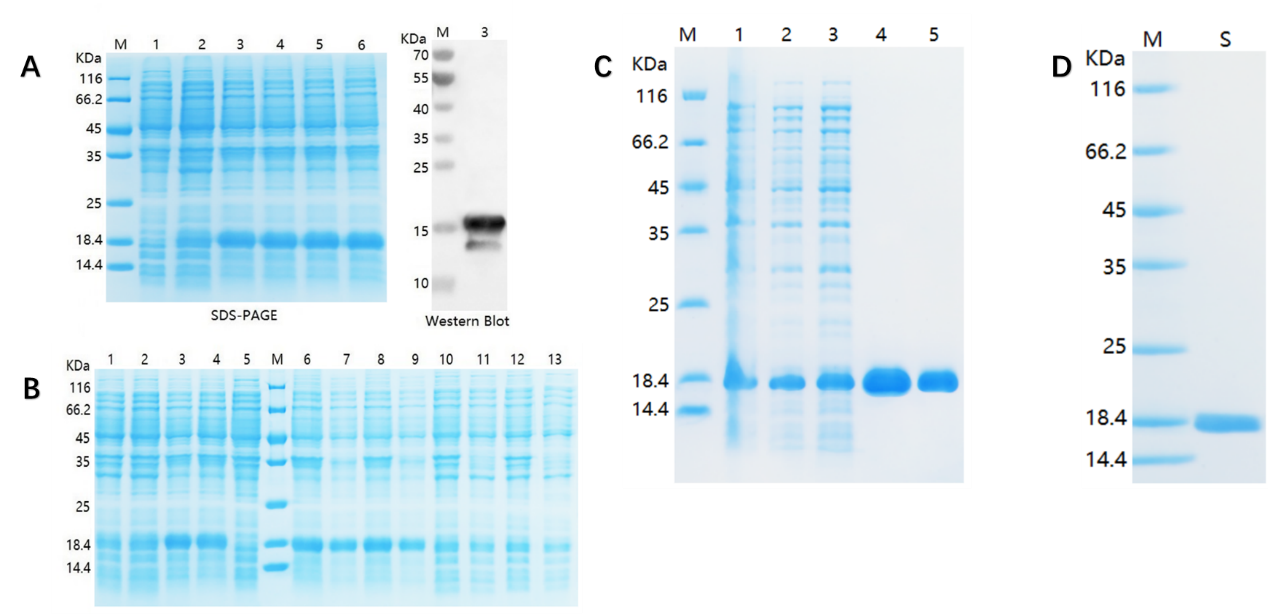


Figure legend: (A) Expression analysis. Lane M: Protein marker; Lane 1: uninduced sample; Lanes 2–6: induced samples. (B) Optimization and solubility analysis. Lane M: Protein marker; Lane 1: Sample induced with 0.2 mM IPTG at 15℃; Lane 2: Sample induced with 1.0 mM IPTG at 15℃; Lane 3: Sample induced with 0.2 mM IPTG at 37℃; Lane 4: Sample induced with 1.0 mM IPTG at 37℃; Lane 5: Uninduced sample; Lane 6: Pellet fraction after induction with 1.0 mM IPTG at 37℃; Lane 7: Supernatant fraction after induction with 1.0 mM IPTG at 37℃; Lane 8: Pellet fraction after induction with 0.2 mM IPTG at 37℃; Lane 9: Supernatant fraction after induction with 0.2 mM IPTG at 37℃; Lane 10: Pellet fraction after induction with 1.0 mM IPTG at 15℃;

Lane 11: Supernatant fraction after induction with 1.0 mM IPTG at 15℃; Lane 12: Pellet fraction after induction with 0.2 mM IPTG at 15℃; Lane 13: Supernatant fraction after induction with 0.2 mM IPTG at 15℃. (C) Purification analysis. Lane M: Protein marker; Lane 1: Pellet after cell lysis; Lane 2: Supernatant after cell lysis; Lane 3: Flow-through fraction; Lane 4: Wash fraction; Lane 5: Elution fraction. (D) Dialysis analysis. Lane M: Protein marker; Lane S: Dialyzed sample.
